# Adult’s veracity judgments of Black and White children’s statements: the role of perceiver and target race and prejudice-related concerns

**DOI:** 10.3389/fpsyg.2023.1177253

**Published:** 2023-07-26

**Authors:** Sarah Zanette, Siham Hagi Hussein, Lindsay C. Malloy

**Affiliations:** ^1^Department of Psychology, Luther College at the University of Regina, Regina, SK, Canada; ^2^Department of Psychology, Faculty of Arts, University of Regina, Regina, SK, Canada; ^3^Faculty of Social Science and Humanities, Ontario Tech University, Oshawa, ON, Canada

**Keywords:** deception, honesty, lying, prejudice, racial bias, veracity judgments, lie-detection, children

## Abstract

**Introduction:**

Seldom has work investigated systematic biases in adults’ truth and lie judgments of children’s reports. Research demonstrates that adults tend to exhibit a bias toward believing a child is telling the truth, but it is unknown whether this truth bias applies equally to all children. Given the pervasiveness of racial prejudice and anti-Black racism in the United States, the current study examined whether adults are more or less likely to believe a child is telling the truth based on the race of the child (Black or White), the race of the adult perceiver (Black or White), and the perceiver’s concerns regarding appearing unprejudiced.

**Methods:**

Using an online data-collection platform, 593 Black and White American adults reviewed fictitious vignettes in which a child denied committing a misbehavior at school (e.g., damaging a laptop). The race of the child in the vignette was manipulated using an AI-generated photo of either a Black child or a White child. After reading each story, participants provided a categorical veracity judgment by indicating whether they believed the child in the story was lying (and therefore committed the misdeed) or telling the truth (and was innocent), as well as rated how honest or deceptive the child was being on a continuous scale. Participants also completed questionnaires assessing their internal (personal) and external (normative) motivations to respond in non-prejudiced ways.

**Results and discussion:**

Results indicated that systematic racial biases occur in adults’ veracity judgments of children’s statements. Both Black and White participants exhibited a truth bias in their veracity judgments of Black children, but not when evaluating the deceptiveness of White children. Consistent with the prejudice-related concerns hypothesis, the observed truth bias toward Black children was moderated by individual differences in participants’ desire to respond without prejudice and whether those motivations stem from external or internal sources. The current findings present novel evidence regarding racial bias and prejudice-related concerns as potential barriers to making veracity judgments of children’s statements and, ultimately, successful lie detection.

## 1. Introduction

Children tell lies for many reasons: One of the most common reasons is to protect themselves from the consequences of their transgressions (Stouthamer-Loeber, 1986; Newton et al., 2000; Wilson et al., 2003). These transgressions are often relatively benign, such as peeking during a guessing-game (e.g., Talwar and Lee, 2002; Bruer et al., 2020; Liu et al., 2022). However, children may also lie in situations in which the consequences are more serious, such as lying to conceal cheating on a test in school (e.g., Zhao et al., 2021), in legal cases for which the child is a suspect (e.g., Redlich Q. et al., 2008) or victim/witness to a crime (e.g., Redlich G. et al., 2008; Dykstra et al., 2022; Price et al., 2022), and even in cases where the child is a victim of abuse or maltreatment (Lyon et al., 2008; Williams et al., 2020). Adults, such as parents, teachers, social workers, and law-enforcement personnel, regularly face the challenge of determining whether a child is being honest or deceptive – also known as *veracity judgments*.

Decades of research has examined how adults assess the veracity of children’s statements, both in developmental (e.g., Talwar and Lee, 2002; Talwar et al., 2006) and legal contexts (e.g., Ross et al., 2003; O’Connor et al., 2023). These studies have primarily focused on issues regarding accuracy (see Gongola et al., 2017 for meta-analysis). While it is important to determine whether adults are *accurate* deception detectors, it is also important to determine whether adults are *biased* deception detectors – since labeling statements as a “lie” regardless of accuracy has meaningful consequences. In the current study, we followed the suggestions of Lloyd et al. (2017) and investigated what factors may contribute to individuals being biased detectors of children’s lies. Specifically, we examined (1) whether adults’ veracity judgments of children’s statements are influenced by the race of the child target, the race of the adult perceiver, or both; and (2) whether such veracity judgments are related to the perceiver’s desire to act – or *appear* to act – in non-prejudiced ways. These are important questions to investigate because deciding whether someone is being honest or deceptive based on prejudicial biases – and not factual evidence – could reduce the accuracy of such judgments and potentially lead to serious consequences.

### 1.1. Truth bias in detecting children’s lies

Extant research has found that adults tend to be more accurate at identifying children’s true statements as true (60% accuracy rate) than they are at identifying children’s false statements as false (49% accuracy rate; see Gongola et al., 2017 for meta-analysis). Furthermore, consistent with the patterns observed in their veracity judgments of other adults (e.g., Levine et al., 1999; Levine, 2014), adults seem biased toward wrongfully labeling children’s false statements as being true (Gongola et al., 2017). One reason for this pattern may be that adults often believe that children are simply unlikely to tell a lie (Quas et al., 2005; Goodman et al., 2006; Talwar et al., 2006). Alternatively, this may be due to a general anchoring effect, in which people tend to believe that social interactions are honest and often fail to sufficiently adjust this assumption when making veracity judgments, resulting in a bias toward their initial position that the person is being truthful (Vrij et al., 2006, 2010; Gongola et al., 2017). Considerable research has documented that adults exhibit a truth bias in their veracity judgments of children’s statements (e.g., Strömwall and Granhag, 2005; Talwar et al., 2006; Talwar et al., 2015; Evans et al., 2016; Saykaly et al., 2017; but see Masip et al., 2004; Crossman and Lewis, 2006; Edelstein et al., 2006), yet it is unknown whether this bias applies equally to all children.

Little work has been done to investigate systematic biases in truth and lie judgments. As a result, it is unclear to what extent particular characteristics of the individual influence whether they are more or less likely to be perceived as honest or deceptive. Previous research has demonstrated that a person’s physical characteristics, such as their facial structure and attractiveness (Zebrowitz et al., 1996; Bond and DePaulo, 2008), can have a substantial effect on whether they are judged to be truthful or dishonest. Race is yet another, perhaps more salient, characteristic that also has the potential to bias adults’ veracity judgments. It could be that factors such as personal prejudices and anti-Black stereotypes about criminality (Plous and Williams, 1995; Welch, 2007; March, 2022) may lead to racial bias in adults’ veracity judgments of children’s reports. This may manifest as a weaker truth bias for Black children compared to White children, or perhaps even as a lie bias, such that adults are more likely to label the statements of Black children as a lie but the statements of White children as the truth. Research is needed to investigate this possibility.

### 1.2. Racial bias

Racial prejudice and anti-Black racism remain a pervasive crisis in the United States. Recent events, such as the murders of George Floyd, Breonna Taylor, Ahmaud Arbery, and other people of color have fueled the ongoing movement to end racial inequality, particularly within the legal system – where racial inequalities have been well-documented (Henderson et al., 1997; Hurwitz and Peffley, 2010; Kovera, 2019). For example, it is a well-established problem that Black people, including Black youth, are overrepresented in United States correctional institutions (U.S. Department of Justice, 2020a, 2020b). Black people are also overrepresented in samples of false confessors (see Najdowski, 2011) – individuals who wrongfully admit to committing a crime, often because of police pressure during interrogation. Najdowski (2011) proposed that the stereotype threat associated with awareness of the Black criminality stereotype (i.e., the idea that Black people are inherently criminal; Plous and Williams, 1995; Welch, 2007; March, 2022) is activated in police interrogations for Black suspects. The activation of this stereotype threat is theorized to lead Black suspects to behave in ways that make them more likely to be judged as being deceptive.

It could be that Black children’s denials of having committed a wrongdoing are less likely to be perceived as honest and are instead more likely to be judged as lying compared to the denials of White children. Adults have also been shown to perceive Black children – particularly Black boys – as older and less childlike than their White same-aged counterparts (Goff et al., 2014). When Black children are victims of abuse, adults tend to perceive them to be more mature and more responsible for that abuse than White children (Bottoms et al., 2004). This is especially concerning since research has shown that older children are judged more harshly and are more likely to be labelled a liar than younger children (Bottoms et al., 2004).

Although we have highlighted the problem of racial inequalities in the justice system, these inequalities begin far earlier in life and occur in a multitude of contexts, particularly within schools. For example, White students are perceived as more compliant than students of color, which decreases the former group’s likelihood of being expelled (Okonofua et al., 2016). In contrast, Black children are more likely than other children to be disciplined in school (Wymer et al., 2022), even when considering factors such as their grades, attitudes, gender, and their conduct in school as perceived by teachers (Rocque and Paternoster, 2011). Research has shown that teachers regard the behaviors of Black children as more hostile than those of White children (Wymer et al., 2022). However, it is largely unknown whether Black children are also perceived to be more or less (dis)honest compared to White children. Evidence from studies of adult targets suggest that race influences adults’ veracity judgments of other adults’ statements – but not necessarily in the pattern one might expect (Lloyd et al., 2017).

In a series of studies conducted by Lloyd et al. (2017), adult participants watched video footage of Black and White college-age individuals describing an acquaintance and were asked to indicate whether they believed the adult in the video was telling the truth or a lie. The results indicated that both Black and White participants judged Black adults as more honest compared to White adults (Lloyd et al., 2017). Participants also exhibited a truth bias for Black adults but showed no such bias for White adults (Lloyd et al., 2017). While this finding seems to contradict our expectations based on the previous discussion regarding the racial inequalities Black people face in the educational and legal systems, analyses examining eye-gaze during the task revealed that while they may have ultimately chosen to judge Black adults as telling the truth, White participants were significantly faster to first fixate on the “lie” response option for Black adults. Together with self-report questionnaire data, Lloyd et al. (2017) interpret these findings as evidence to suggest that the tendency for White participants to label Black targets as more truthful than White targets was influenced by their desire to appear unprejudiced toward Black people. In other words, White participants’ initial judgment of Black targets is that they are lying, but this judgment is then overcome by subsequent processing involving the desire to act in non-prejudiced ways (Lloyd et al., 2017).

Though the findings obtained by Lloyd et al. (2017) suggest that adults demonstrate a truth bias based on race when judging the veracity of *adults’* statements, it is largely unknown whether a similar pattern occurs when judging the veracity of *children’s* statements, since no study (to the best of our knowledge) had examined this question. However, recent work by O’Connor et al. (2023) sheds light on a related question. In their study, a sample of primarily (89%) White adults in the UK provided trait-honesty ratings of Black and White children based on a single photograph of the child and were found to explicitly rate Black children as more honest than White children. Similarly, the study found that adults rated children’s (fictitious) testimony of physical abuse as more honest and were more likely to render a guilty verdict for the accused when the child alleging the abuse was Black (79%) compared to when the child alleging abuse was White (69%). These findings may initially suggest the existence of a stronger truth bias for Black children than for White children. However, as with Lloyd et al. (2017), the findings O’Connor et al. (2023) obtained using implicit measures of racial bias contradict this interpretation.

Using the Implicit Association Test (IAT; Nosek et al., 2007), O’Connor et al. (2023) found that adults were implicitly biased to associate White children more strongly with honesty compared to Black children, and that greater implicit racial bias predicted less trust in the child’s testimony and a lower likelihood of convicting the accused of abusing the child. As O’Connor et al. (2023) argue, these results are concerning as they suggest that adults hold implicit biases regarding the honesty of children based on race and that these biases may affect how they appraise case details and render verdicts.

While the findings from both O’Connor et al. (2023) and Lloyd et al. (2017) offer important insights to the complex and potentially dangerous impact of race on veracity judgments, significant gaps in our understanding remain. Notably, O’Connor et al. (2023) examined children’s statements in the context of being a victim of harm (physical abuse) but it is unknown whether these findings generalize to other contexts where deception may occur, such as when the child is the one accused of committing a wrongdoing. Given the potential implications in contexts such as the legal and educational systems (as discussed above), it is important to understand whether adults are biased in their veracity judgments of Black and White children denying misbehavior or misconduct.

### 1.3. The current study

The current study investigated potential racial bias in adults’ veracity judgments using (fictitious) vignettes of an authority figure (i.e., teacher) interviewing a child (age 7) who denies having committed a misbehavior. A sample of Black and White adult participants reviewed the vignettes (two total) and provided two types of veracity judgments for each child: (1) a *categorical* truth-lie judgment (i.e., is the child lying or telling the truth?) and a (2) *continuous* deception rating (i.e., ratings of how honest or deceptive the child is being on a 10-point Likert scale). To determine whether adults perceive Black children as more or less deceptive than White children, the race of the child in each vignette was manipulated using a photo of either a Black or White (randomized within-subjects) girl or boy (randomized between-subjects).

We offer two competing hypotheses regarding the expected direction of racial bias in veracity judgments: Consistent with our earlier discussions of the prevalent stereotype that Black people are inherently criminal (Plous and Williams, 1995; Welch, 2007; March, 2022) and of prejudicial attitudes contributing to adults’ perceptions of Black children as more mature and more responsible for their transgressions compared to White children (Bottoms et al., 2004), the first hypothesis is that Black children will be rated as *more deceptive* than White children. However, extant research also suggests that participants may be influenced by a desire to avoid appearing or acting prejudiced, leading them to inflate their positivity toward Black people (Crandall et al., 2002). As Lloyd et al. (2017) argues, *prejudice-related concerns* may lead individuals to avoid labeling Black people (relative to labeling White people) as liars. Therefore, the second, opposing hypothesis is that participants’ prejudice-related concerns will lead to Black children being rated as *less deceptive* than their White counterparts.

Previous research has shown that the degree of one’s intrinsic and extrinsic motivations to respond without prejudice are related to actual expressions of prejudice and racial bias (Plant and Devine, 1998; Devine et al., 2002; Butz and Plant, 2009; Lloyd et al., 2017). Thus, we examined whether participants’ prejudice-related concerns influenced their veracity judgments using self-report measures of internal (personal) and external (normative) motivation to respond in non-prejudiced ways (Plant and Devine, 1998). We also investigated whether prejudice-related concerns may have differentially influenced Black and White participants’ veracity judgments of children’s statements. For example, White adults may show a stronger truth bias when judging Black children, perhaps in part due to an increased saliency of social norms regarding avoiding racial prejudice against Black people (Plant and Devine, 1998; Crandall et al., 2002; Bergsieker et al., 2010; Kunstman et al., 2013; Mendes and Koslov, 2013; Rozmann and Nahari, 2021). On the other hand, factors such as ingroup favoritism (Turner et al., 1979; Tajfel, 1982) may result in Black people showing a stronger truth bias toward Black children. We begin to address the potential role of the race of the child in question, the race of the adult making the veracity judgment, and the adult’s prejudice-related concerns in the current study.

## 2. Materials and methods

### 2.1. Participants

Sample size was determined by *a priori* power analyses to detect a small effect (0.12), with power set at 0.85 and *α* = 0.05, conducted in G*Power 3.1.9 (Faul et al., 2009). Based on power analyses of the mixed model analysis of variance (ANOVA) and hierarchical linear and logistic regressions needed, it was determined that 592 participants would be needed to detect a small effect (chosen based on Lloyd et al., 2017). Including a buffer for participants who fail attention checks, we sought to recruit 600 participants for this study using Prolific, an online crowdsourcing research platform. A total of 609 Black and White jury-eligible United States citizens (aged 18+, no felonies, English fluent) participated. Data was removed from participants who stated they had felony convictions, failed attention checks, took less than 2 min to complete the study, or provided the same score on every item of every measure (suggesting they erroneously selected responses). A total of 16 participants were excluded from the study based on these criteria, resulting in a final sample of 593 participants (50% Black, 50% White) ranging from 18 to 79 years of age (*M*_age_ = 35.89, *SD* = 13.13). Roughly half (49%) of participants were male, 49% female, and less than 2% (*n* = 11) identified as nonbinary or genderfluid. Participants resided in a variety of geographical regions across the United States. Based on the geographic regions identified by the United States Census Bureau (2021), 48% of participants were from the South, 21% from the Midwest, 16% from the Northeast, and 15% from the West.

### 2.2. Procedure

This study was reviewed and approved by the University of Regina Research Ethics Board. Participants accessed the study through Prolific, which then redirected them to Qualtrics, where they provided written informed consent and completed the study procedures. Participants were told that the purpose of the study was to explore adults’ accuracy when judging the reports of children. To reduce the potential for demand characteristics having an influence on their responses, participants were not informed of the racial bias component of the study goals until after they had completed the study (during debriefing).

Participants were first asked to provide basic demographic information. Next, they completed a veracity judgment task and answered questionnaires regarding their “personal beliefs” (i.e., their motivation to respond without prejudice). The order of tasks was counterbalanced so that half of participants completed the veracity judgment task *before* the questionnaires, while the other half did the veracity judgment task *after* the questionnaires. This was done to reduce the potential for demand characteristics and priming effects that may arise due to task order. At the end of the study, participants were fully debriefed on the purpose and goals of the study.

### 2.3. Veracity judgment task

During the veracity judgment task, participants were asked to review two fictitious vignettes, each outlining a scenario where a teacher suspects a 7-year-old of committing a wrongdoing in school (cheating on a spelling test or damaging a laptop). In each scenario, the teacher has reasons to suspect that the child is guilty of the transgression, but the evidence is unclear. When the teacher asks the child about it, the child denies the misbehavior. The vignette was intentionally written so that it is unclear whether the child committed the misdeed and is lying about having done so, or whether the child is innocent and is being truthful in their denial of the wrongdoing. The race of the child in each story was experimentally manipulated by presenting a photo of either a Black child or a White child (artificially created using Generated Photos, n.d.) alongside each vignette. The child’s name in the vignette was also changed to one that is stereotypically associated with the targeted race and therefore may increase the saliency of the child’s race.

To maximize the statistical power of race-related hypotheses tests, the race of the child was experimentally manipulated within-subjects, whereas the gender of the child was manipulated between-subjects. Participants were therefore randomly assigned to review and provide veracity judgments for one Black boy and one White boy, or one Black girl and one White girl. The order in which participants reviewed each vignette was counterbalanced and evenly distributed among White and Black participants.

After reviewing each vignette, participants gave two types of veracity judgments for each child. First, participants provided a *categorical* veracity judgment by indicating whether they believed the child in the story was lying (and therefore committed the misdeed) or telling the truth (and was innocent). Participants were not given the option to skip this question or indicate that they were “unsure” and did not know whether the child was being honest or deceptive. This was intentional, as we wanted to mimic real-world contexts where adults are forced to make veracity judgments regarding children’s statements in the face of ambiguous or unclear evidence. However, the use of a binary outcome measure has its limitations due to decreased variability in potential responses. Furthermore, the nature of the vignettes is such that the child is given multiple opportunities to either lie or tell the truth when speaking with the teacher. To overcome these limitations, participants also rated how honest or deceptive the child was on a 10-point Likert scale, from *not at all deceptive* (1) to *very deceptive* (10).

### 2.4. Motivation to respond without prejudice

The Internal and External Motivation to Respond Without Prejudice scales (Plant and Devine, 1998) were used to measure participants’ personal (internal) and normative (external) motivations to respond without prejudice. As described by Devine and colleagues, “internal motivation to respond without prejudice arises from internalized, personally important nonprejudiced beliefs (i.e., the self sets the standard against which one’s prejudice-relevant responses are evaluated).” In contrast, “external motivation to respond without prejudice derives from a desire to avoid negative reactions from others if one were to respond with prejudice (i.e., others impose the standard against which one’s prejudice-relevant responses are evaluated)” (Devine et al., 2002, p. 836).

The internal motivation scale (IMS) contains 5 items, such as “*I attempt to act in non-prejudiced ways toward Black people because it is personally important to me*.” The external motivation scale (EMS) also contains 5 items, such as “*because of today’s politically correct standards I try to appear non-prejudiced toward Black people*.” Participants responded to each item using a 9-point Likert scale, ranging from-4 (*strongly disagree*) to +4 (*strongly agree*). Responses were then averaged to create two distinct, but related, measures of participants’ motivation to appear unprejudiced (Plant and Devine, 1998). The EMS and IMS subscales achieved high internal consistency within this study (*α* = 0.88 and *α* = 0.85, respectively).

## 3. Results

We first used the McNemar test of paired-samples proportions and one-sample chi-square tests to examine whether adults demonstrate a racial bias in their categorical truth-lie judgments of children’s statements. Next, we conducted a mixed model analysis of variance (ANOVA) to determine whether adults’ continuous deception ratings differ based on their own race, the race of the child in the vignettes, or both. Lastly, we used hierarchical logistic and linear regression analyses to examine whether participants’ prejudice-related concerns (i.e., internal and external motivations to not appear prejudiced) are related to their veracity judgments of Black children. Initial analyses indicated no significant effects of the order in which participants completed the veracity judgment task (before or after completing the questionnaires) or the order in which they viewed each child in the vignettes (Black child or White child first). Order variables were therefore removed from analyses and the more parsimonious results are presented here.

### 3.1. Effects of the child’s race on veracity judgments

Of primary interest was whether adults demonstrate a racial bias when judging the veracity of Black and White children’s statements. We examined participants’ *categorical* truth-lie judgments (i.e., is the child lying or telling the truth?) and (2) *continuous* deception ratings (i.e., ratings of how deceptive the child is being on a 10-point Likert scale) separately to investigate this question.

#### 3.1.1. Categorical truth-lie judgments

**Figure 1:**
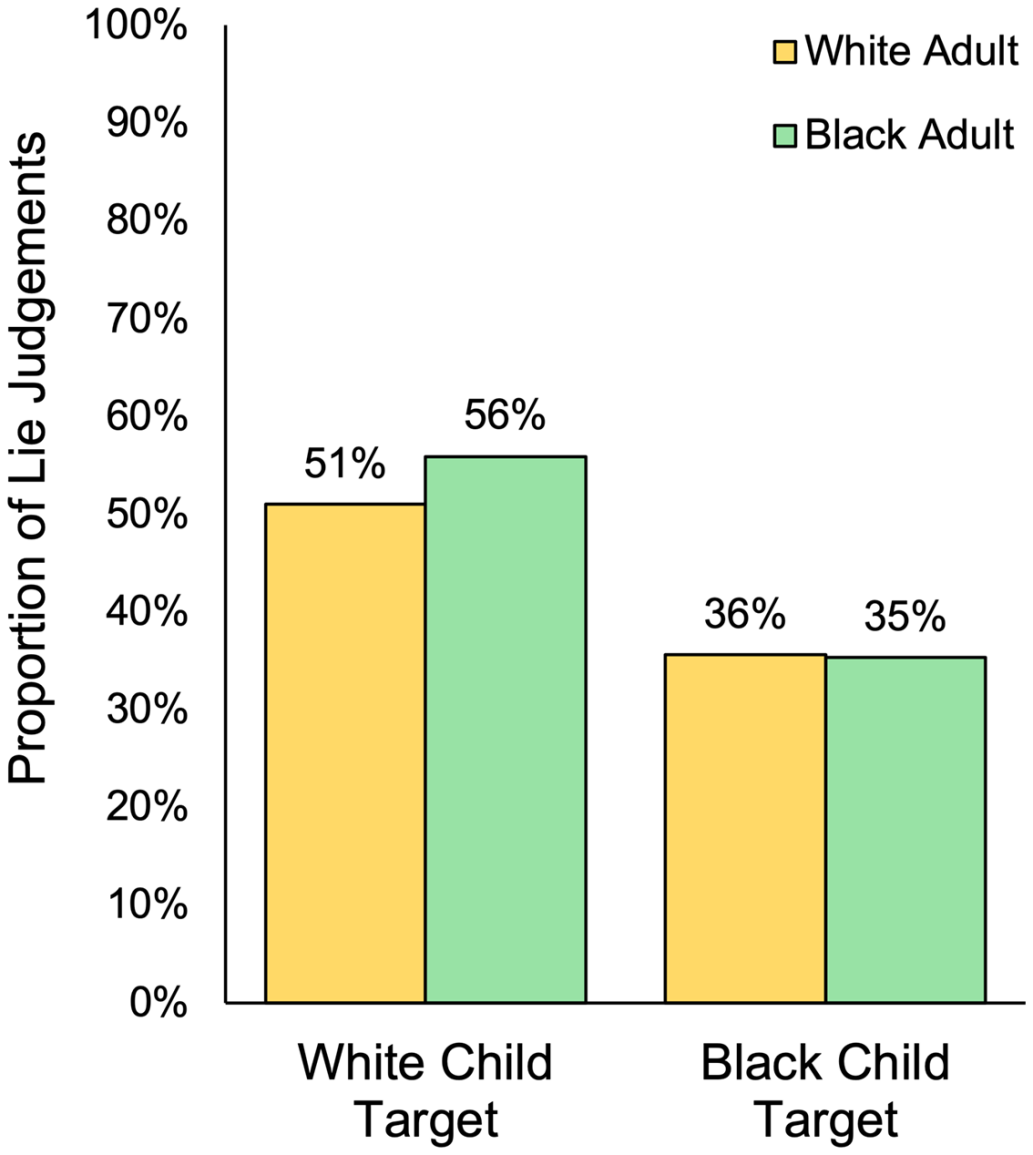
Differences in categorical truth-lie judgments based on the race of the child target and the race of the adult perceiver.

A McNemar’s test with continuity correction was conducted separately for Black participants and White participants to determine if there was a difference in the proportion of truth and lie judgments based on the race of the child. As shown in Figure 1, results revealed that, among White participants, the proportion of lie judgments was significantly greater for White children (51% labelled as lying) compared to Black children (36% labelled as lying), *χ*^2^ = 14.89, *p* < 0.001, mean difference in proportions = 0.15 (95% CI [0.08, 0.23]). Similarly, Black participants also gave more lie judgments to White children (56% labelled as lying) compared to Black children (35% labelled as lying), *χ*^2^ = 27.07, *p* < 0.001, mean difference in proportions = 0.21 (95% CI [0.13, 0.28]). An examination of confidence intervals indicates that the proportion of the difference in lie judgments given to White children compared to Black children did not significantly differ based on the race of the participant, *p* > 0.05. In other words, White participants and Black participants demonstrated similar levels of bias in categorical truth-lie judgments based on the child’s race (Figure 1).

Follow-up one-sample chi-square tests indicated that the categorical veracity judgments of White children did not significantly differ from chance (50%), indicating that – regardless of their own race – participants were no more likely to label White children as lying (54%) as they were to label them as telling the truth (46%), *χ*^2^ (1, *N* = 593) = 2.84, *p* = 0.092. In contrast, they were significantly less likely to label Black children as lying (36%) and more likely to label them as telling the truth (64%), *χ*^2^ (1, *N* = 593) = 49.81, *p* < 0.001. Taken together, these findings suggest that Black adults and White adults exhibit a truth bias in their categorical truth-lie judgments of Black children, but they show no such bias toward White children.

#### 3.1.2. Continuous deception ratings

Utilizing participant’s continuous deception ratings (higher scores = more deceptive) as the dependent variable, we conducted a 2 (participant race) × 2 (child race) mixed model analysis of variance (ANOVA) to determine whether continuous veracity judgments differ based on the race of the participant (between-subjects) or the race of the child (within-subjects) in the vignettes.

**Figure 2:**
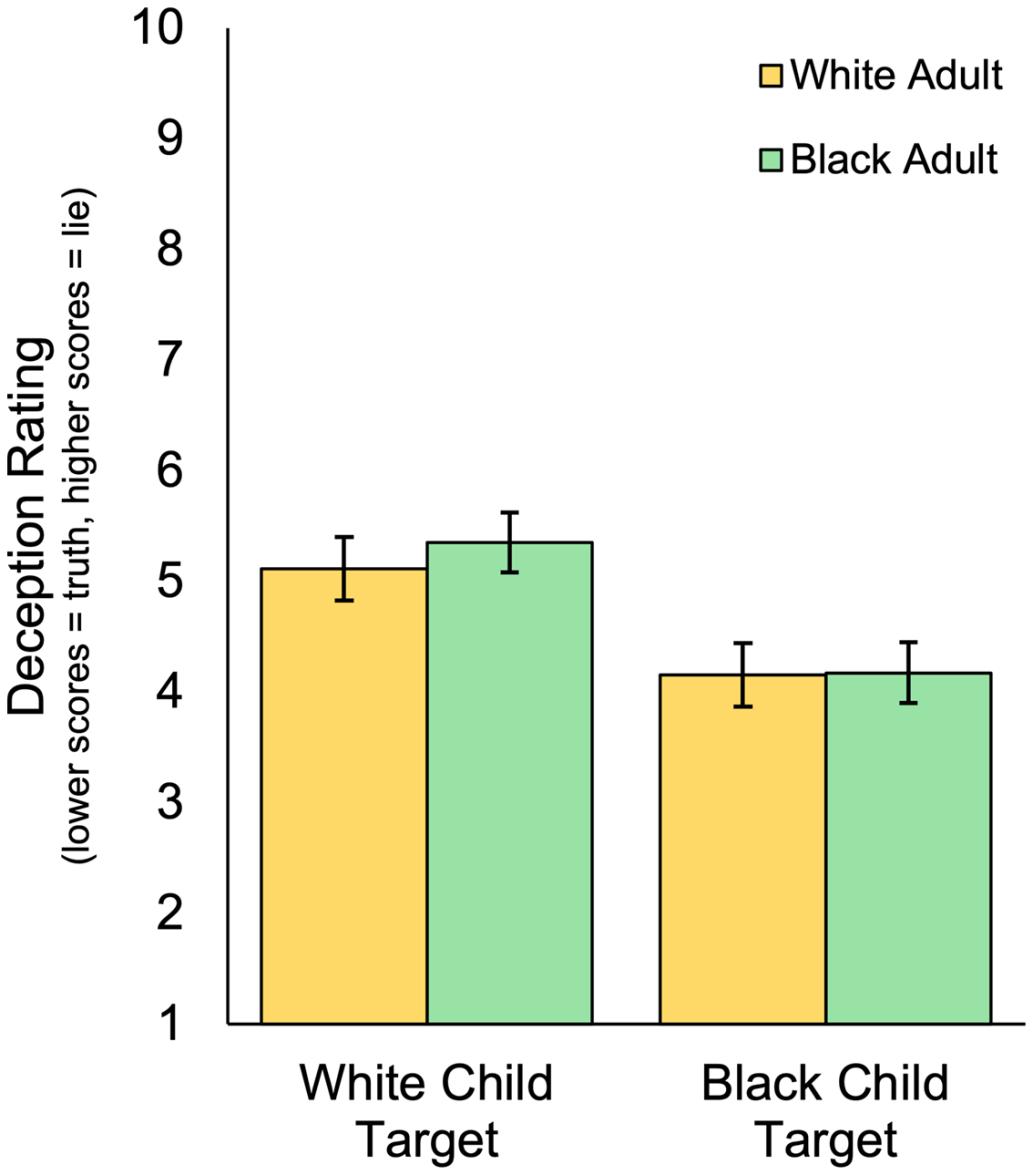
Differences in average continuous deception ratings as a function of the race of the child target and the race of the adult perceiver. Error bars represent 95% confidence intervals.

Consistent with our findings obtained using the categorical truth-lie judgments, results revealed a significant main effect of the child’s race on continuous deception ratings, such that White children (*M* = 5.23, *SD* = 2.52, 95% CI [5.03, 5.44]) were rated as more deceptive than Black children (*M* = 4.16, *SD* = 2.39, 95% CI [3.97, 4.36]) by an average of 1.07 points (95% CI of the difference [0.81, 1.33]), *F*(1, 591) = 65.28, *p* < 0.001, $\eta_{p}^{2}\;$= 0.099. Neither the main effect of participant race, *F*(1, 591) = 0.73, *p* = 0.394, $\eta_{p}^{2}\;$= 0.001, nor the interaction between participant race and child race, *F*(1, 591) = 0.71, *p* = 0.399, $\eta_{p}^{2}\;$= 0.001, were significant (Figure 2). Thus, just as with categorical truth-lie judgments, both Black adults and White adults gave Black children lower deception ratings compared to White children.

### 3.2. Prejudice-related concerns and veracity judgments of Black children

We next examined how prejudice-related concerns may have differentially influenced Black and White participants’ judgments of whether the child in the vignette was telling the truth or a lie. If, as we hypothesized, the observed truth bias toward Black children was driven at least in part by prejudice-related concerns, then the magnitude of this bias should be predicted by individual differences in participants’ internal and external motivation to not appear prejudiced (i.e., their IMS and EMS scores). In line with Lloyd et al. (2017), we regressed the deception ratings for Black children on participant race (coded as 0 = White and 1 = Black), IMS score, EMS score, the interaction terms of EMS × participant race and IMS × participant race, and we entered participants’ deception ratings for the White children as a covariate to control for individual differences in participants’ overall willingness to believe a child is lying versus being truthful. We conducted separate hierarchical regressions for each of the types of veracity judgments obtained: a logistic regression was used to examine the categorical truth-lie judgments (Table 1) and a linear regression was used to analyze the continuous deception ratings (Table 2). The order of variable entry was identical across analyses: we entered the covariate alone on the first step, all main effects on the second step, and the interactions on the third step.

**Table 1:** Hierarchical logistic regression results for prejudice-related concerns predicting categorical truth-lie judgments (0 = truth, 1 = lie).

	*χ* ^2^	*R* ^2^	*ΔR* ^2^	*B*	*SE B*	Wald	Odds ratio	95% CI for odds ratio
**Model 1**	7.36**	0.02	0.02					
Constant				−0.37**	0.12	9.94	0.69	
Deception rating: white child				−0.48**	0.18	7.27	0.62	[0.44, 0.88]
**Model 2**	6.64	0.03	0.02					
Constant				−0.01	0.20	<0.01	0.99	
Deception rating: white child				−0.48**	0.18	7.27	0.62	[0.44, 0.88]
Participant race				−0.07	0.18	0.14	0.93	[0.66, 1.33]
IMS				−0.15*	0.06	6.56	0.86	[0.77, 0.97]
EMS				<0.01	0.05	<0.01	1.00	[0.91, 1.10]
**Model 3**	5.73	0.05	0.01					
Constant				0.35	0.26	1.83	1.42	
Deception rating: white child				−0.50**	0.18	7.65	0.61	[0.43, 0.87]
Participant race				−0.67*	0.32	4.32	0.51	[0.27, 0.96]
IMS				−0.31**	0.09	11.39	0.74	[0.62, 0.88]
EMS				<−0.01	0.07	<0.01	1.00	[0.86, 1.15]
Participant race × IMS				0.28*	0.12	5.56	1.33	[1.05, 1.68]
Participant race × EMS				<0.01	0.10	<0.01	1.00	[0.83, 1.22]
**Overall model**	19.72**	0.05						

Black participants serve as the reference group for all steps: Participant Race coded as White = 0, Black = 1. IMS = internal motivation to respond without prejudice score; EMS = external motivation to respond without prejudice score; CI = confidence interval. *p < 0.05, **p < 0.01.

**Table 2:** Hierarchical linear regression results for prejudice-related concerns predicting continuous deception scores.

	*R* ^2^	*ΔR* ^2^	*B*	95% CI for *B*	*SE B*	*β*
**Model 1**	0.02	0.02***				
Constant			3.47***	[3.02, 3.92]	0.23	
Deception rating: white child			0.14***	[0.06, 0.22]	0.04	0.15
**Model 2**	0.04	0.02*				
Constant			3.98***	[3.40, 4.56]	0.30	
Deception rating: white child			0.13**	[0.06, 0.21]	0.04	0.14
Participant race			−0.02	[−0.42, 0.37]	0.20	−0.01
IMS			−0.20**	[−0.32, −0.07]	0.07	−0.12
EMS			0.04	[−0.07, 0.15]	0.06	0.03
**Model 3**	0.05	0.01				
Constant			4.36***	[3.69, 5.03]	0.34	
Deception rating: white child			0.13**	[0.05, 0.21]	0.04	0.14
Participant race			−0.66	[−1.37, 0.05]	0.36	−0.14
IMS			−0.36***	[−0.55, −0.17]	0.10	−0.23
EMS			0.03	[−0.13, 0.19]	0.08	0.02
Participant race × IMS			0.29*	[0.04, 0.55]	0.13	0.19
Participant race × EMS			0.02	[−0.20, 0.23]	0.11	0.01
**Overall model**	*F* = 4.53***					

Black participants serve as the reference group for all steps: Participant Race coded as White = 0, Black = 1. IMS = internal motivation to respond without prejudice score; EMS = external motivation to respond without prejudice score; CI = confidence interval. *p < 0.05, **p < 0.01, ***p < 0.001.

In both cases, the overall model significantly predicted 5% of the variance in adults’ deception ratings of Black children. The results obtained from the hierarchical logistic (Table 1) and hierarchical linear (Table 2) regressions diverged from one another in terms of which individual steps in the model significantly contributed to the model over and above the contributions of prior steps (the logistic regression found only step 1 to be independently significant, whereas the linear regression found steps 1 and 2 to be independently significant). However, the results of the final overall models were largely consistent regardless of the type of veracity judgment examined (categorical or continuous) and are thus discussed jointly.

The following results were obtained from both sets of analyses unless otherwise explicitly stated. Participants’ veracity judgments of White children significantly predicted their veracity judgments of Black children. The positive direction of the coefficients in the models indicates that participants who rated White children as telling a lie and gave higher deception ratings for White children also did so for the Black children (see Tables 1, 2).

**Figure 3:**
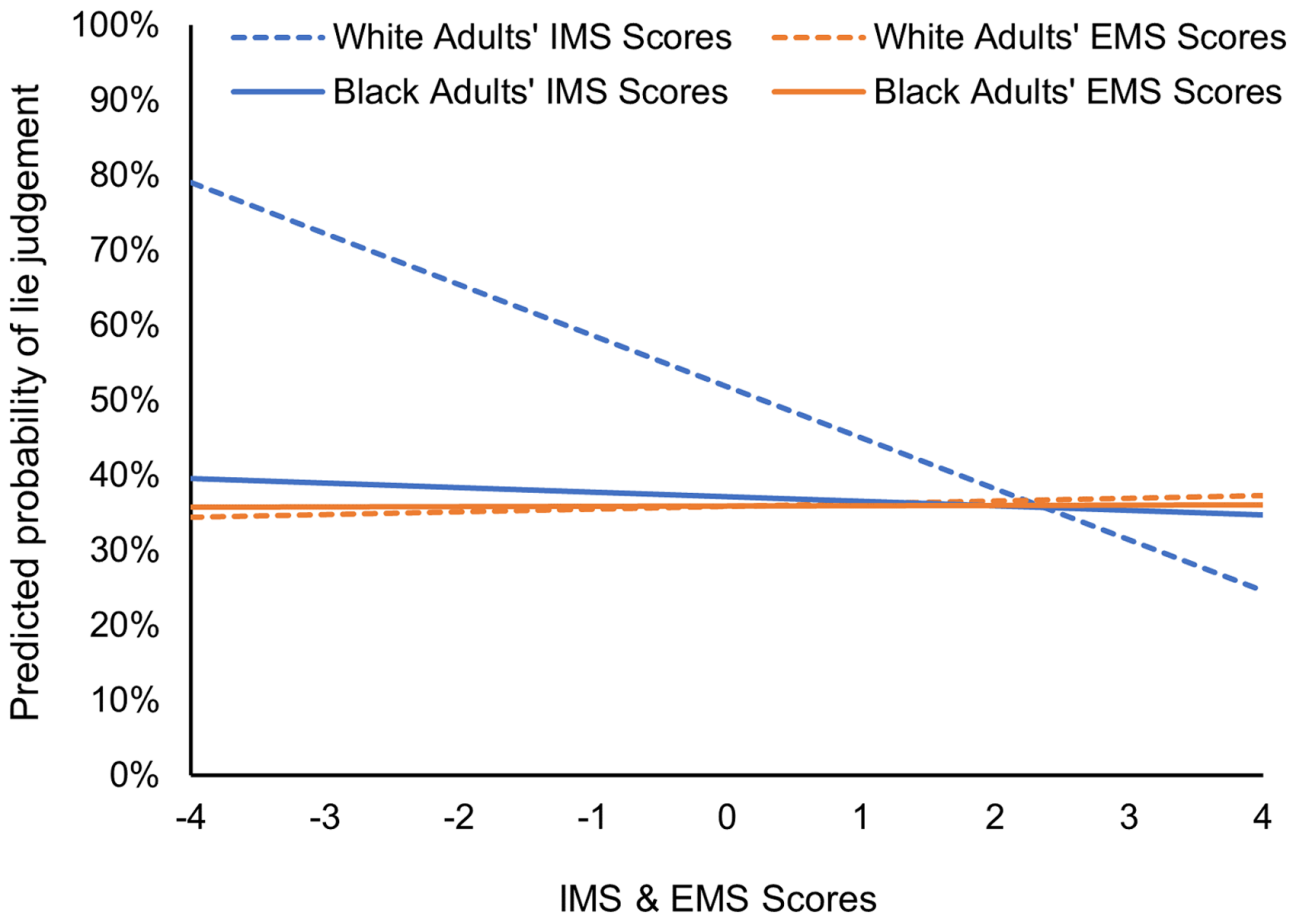
Predicted probability of a lie (versus truth) response given to Black children as a function of the race of the adult perceiver and their internal (IMS scores) and external (EMS scores) motivation to respond without prejudice. Results are based on the final logistic regression model (Table 1) conducted on adults’ categorical truth-lie judgments of Black children after controlling for their truth-lie judgments for White targets.

Participants’ IMS scores, but not EMS scores, were found to be significant predictors of adults’ veracity judgments of Black children. However, these main effects must be interpreted in conjunction with the two interaction terms examined: IMS × participant race and EMS × participant race. Across analyses, neither the main effect of EMS scores nor the interaction of EMS × participant race was found to be significant (all *p*s > 0.05; see Tables 1, 2). Thus, it appears that for both White adults and Black adults, their degree of external motivation to respond without prejudice is not significantly related to their veracity judgments (categorical or continuous) of Black children. In contrast, a significant IMS × participant race interaction term was observed, indicating that the relation between IMS and veracity judgments of Black children depends on the race of the adult participant. Simple slopes analysis revealed that there was a statistically significant negative relationship between IMS scores and veracity judgments among White adults (*p*s < 0.01), but not Black adults (*p*s > 0.05). Regarding categorical truth-lie judgments (Table 1), an odds ratio of 0.51 suggests that for every 1-point increase in IMS scores, White participants are nearly half as likely to judge a Black child as telling a lie (Figure 3). Similarly, increases in White participants’ internal motivation to respond without prejudice are associated with significantly lower deception ratings of Black children (Table 2).

**Figure 4:**
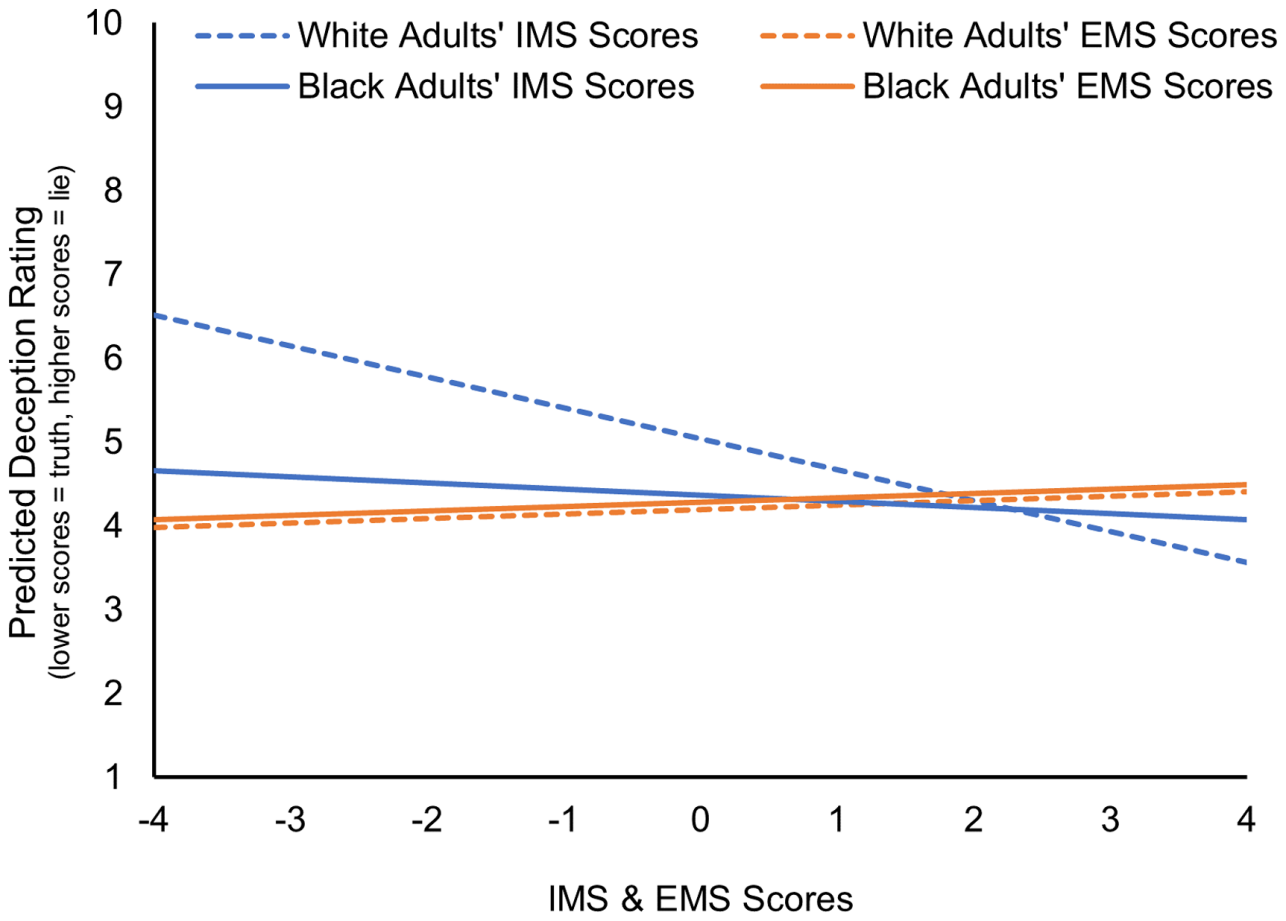
Predicted deception ratings given to Black children as a function of the race of the adult perceiver and their internal (IMS scores) and external (EMS scores) motivation to respond without prejudice. Results are based on the final linear regression model (Table 2) conducted on adults’ continuous deception ratings of Black children after controlling for their deception ratings for White targets.

Examining the predicted veracity judgments generated by the logistic and linear regression models (Figures 3, 4, respectively) reveals that White adults who score in the mid to high range of internal motivation to respond without prejudice and Black participants of any IMS or EMS score all seem to exhibit a truth bias in their veracity judgments of Black children (below 50% probability of a lie judgment in Figure 3 and below the deception rating midrange of 5–6 in Figure 4). In contrast, White adults who score very low in internal motivation to respond without prejudice do not show such bias.

## 4. Discussion

The current study examined whether adults exhibit racial bias in their veracity judgments of children’s reports. Our key goals were to determine if adults’ judgments of whether children are being deceptive about a suspected misbehavior at school differ based on the race of the child (Black or White), the race of the adult perceiver (Black or White), and the perceiver’s motivations to appear unprejudiced. Our findings revealed that systematic race-based biases occur in adults’ veracity judgments of children’s statements, along with evidence to suggest that such biases are related to the perceiver’s prejudice-related concerns.

### 4.1. Race differences in truth bias

In the current study, White children were more likely to be categorically labelled a liar (versus a truth-teller) compared to Black children. This finding is consistent with the race-based biases Lloyd et al. (2017) reported to occur when adults made veracity judgments of other adults. We also found that participants’ categorical veracity judgments of White children did not significantly differ from chance – meaning that participants were no more likely to label White children as lying (54%) as they were to label them as telling the truth (46%). In contrast, participants were significantly less likely to label Black children as lying (36%) and more likely to label them as telling the truth (64%). The same pattern emerged when examining the continuous measure of veracity judgments: Similar to O’Connor et al. (2023), who showed that White adults explicitly rate Black children as being higher in trait-honesty than White children, we found that participants of both racial groups (Black adults and White adults) gave lower deception ratings to Black children compared to White children, indicating that Black children were perceived as being more honest in their denials of wrongdoing compared to White children. Taken together, our findings suggest that adults exhibit a truth bias in their veracity judgments of Black children, but not White children. There are several ways this finding may be interpreted.

One possible interpretation could be due to the stimuli used in the current study: Perhaps the vignettes performed as intended and created an ambiguous situation where it was unclear whether the child committed the misbehavior that they were accused of – resulting in random guessing and chance-level responding across participants. From this perspective, when adults were trying to assess whether a White child was being honest or deceptive, it could be that they felt like they did not have enough information to make a clear veracity judgment one way or the other but they were forced to make such a judgment because no neutral response option was provided (e.g., “*I do not know*” or “*unsure*”). However, additional research is needed to properly assess this hypothesis. Moreover, though this may explain why adults were just as likely to label a White child as telling the truth versus telling a lie, their veracity judgments of Black children tell a different story: In both their categorical veracity judgments and continuous deception ratings of Black children, participants showed a clear bias toward believing that Black children were telling the truth and were being more honest than they were being deceptive. The fact that the truth bias was observed with Black children, but not White children, suggests that knowledge of the child’s race – specifically that they are Black – was enough information for participants to tip the metaphorical scales toward reporting that they believed the child was telling the truth instead of a lie. While this may be interpreted as an unfair advantage granted to Black children but not White children, recall that the truth bias is a well-documented phenomenon (e.g., Strömwall and Granhag, 2005; Talwar et al., 2006, 2015; Evans et al., 2016; Gongola et al., 2017; Saykaly et al., 2017) and past studies have found that the responses that adults give to children are indeed truth-biased. This is best reflected in the current study by the responses that adults made of Black children. From this perspective, what appears counter-normative is not how people responded to Black children, but the absence of a truth bias observed when rating White children.

One potential explanation for why adults demonstrated a truth bias for Black children but not White children could concern the harmful Black criminality stereotype, which falsely contends that Black people are inherently criminal (Plous and Williams, 1995; Welch, 2007; Levinson et al., 2010; Goff et al., 2014; Todd et al., 2016; March, 2022). It could be that White and Black participants are aware of the Black criminality stereotype and attempt to combat its harmful effects by underestimating their perceptions of dishonesty (or inflating their perceptions of honesty) regarding Black children. Additional research is needed to elucidate whether adults’ veracity judgments are indeed a product of their desire to combat the anti-Black criminality stereotype and if so, determine whether they are consciously aware of this source of bias in their veracity judgments or if it occurs on a conscious or subconscious level. Although examining knowledge and beliefs regarding the anti-Black criminality stereotype was not a goal of the current study, we did examine whether prejudice-related concerns may have differentially influenced Black and White participants’ veracity judgments of children’s statements.

### 4.2. Prejudice-related concerns

In findings consistent with the prejudice-related concerns hypothesis, the observed truth bias toward Black children was moderated by individual differences in whether participants were motivated to respond without prejudice and whether those motivations stem from external or internal sources. We found that, regardless of their own race, participants’ level of external motivation to respond without prejudice was not a significant factor in their judgments of the deceptiveness of Black children. This suggests that participants seemed unconcerned about whether their veracity judgments of Black children would be perceived as prejudiced. This effect is unsurprising given that participants completed this study online and were anonymous. It is possible that we would have observed a significant effect of externally motivated prejudice-related concerns if participants completed the study in-person in the presence of a research assistant or other participants (Maeder et al., 2018).

On the other hand, for White adults only, participants’ *internally* motivated prejudice-related concerns were significantly negatively related to their deception ratings of Black children. That is, White adults with greater internal motivation to respond without prejudice rated Black children as less deceptive (more honest) compared to participants with lower internal motivations, suggesting that the truth bias White adults exhibit toward Black children may be driven (at least in part) by participants’ inner desires to respond without prejudice. This finding is consistent with those obtained by Lloyd et al. (2017) regarding the veracity judgments that adults give to other adults. However, it is also important to recognize once again that such a truth bias in veracity judgments of children’s statements is generally normative (e.g., Strömwall and Granhag, 2005; Talwar et al., 2006, 2015; Evans et al., 2016; Gongola et al., 2017; Saykaly et al., 2017). Notably, only very low levels of internal motivation to respond without prejudice among White adults were associated with neutral, chance-level veracity judgments. In contrast, all other patterns – internal motivation scores in the mid to high range of among White participants and Black participants of any internal or external motivation scores – were associated with a truth bias in their veracity judgments of Black children.

Caution is warranted regarding interpreting the relation (or lack thereof) between Black participants’ internal and external motivation to respond without prejudice scores and their veracity judgments of Black children. Black participants responded to the motivation to respond without prejudice measures as in-group members. Thus, the responses of a White participant (a potential actor of prejudice) may be qualitatively distinct from those of a Black participant (a potential victim of prejudice). Although Black adults may still demonstrate a prejudice toward other Black people (David et al., 2019), it is possible that the IMS/EMS scales may be capturing different motivations or desires to respond without prejudice for these participants. It would be beneficial for future research to qualitatively assess this possibility.

### 4.3. Limitations and future directions

A potential limitation of this study is the use of vignettes instead of, for example, video footage of children denying having committed a misbehavior or participants witnessing a live mock trial. Although, O’Connor et al. (2023) deployed a similar methodology to the current study, where participants rated the honesty of Black and White children after reading vignettes describing a legal scenario, it remains possible that the vignettes may not have triggered the same biases that would otherwise emerge in the real-world. However, while responses to vignettes may be imperfect guides to actual behavior (Malloy et al., 2014), they are commonly used in deception research (e.g., Redlich G. et al., 2008; Popliger et al., 2011; Zanette et al., 2020; O’Connor et al., 2023) because they allow researchers to systematically test the effects of key variables of interest and may help circumvent challenges associated with socially desirable responding. It will be important for future work in this area to assess the relation between the race of the child and adult’s veracity judgments both in the field and in the laboratory (Malloy et al., 2014).

The current evidence suggests that adults are more likely to perceive Black children as being less deceptive (more honest) than White children and that concerns regarding acting in non-prejudiced ways may contribute to a truth bias toward Black children but not White children, at least when it comes to elementary-aged children’s simple denials of minor transgressions in a school setting. However, it is important to note that this finding is inconsistent with many inequalities present in the real-world that place Black children at a disadvantage compared to White children in educational and legal contexts. For example, Black elementary school students have been shown to be more likely to experience disciplinary practices from their teachers (Wymer et al., 2022) and receive more severe disciplinary actions, such as school suspensions and expulsions (McFadden et al., 1992; Rocque and Paternoster, 2011) compared to White children. The current study’s findings that Black children are perceived to be more truthful than White children may therefore be due to limitations regarding the external validity of our study design, including potential issues with socially desirable responding.

Another factor to consider is that participants were aware that their veracity judgments were given for research purposes and therefore did not directly impact children in the real world. Moreover, the design of the current study meant that there were only two trials per participant. As highlighted by Levine et al. (2022), there may be idiosyncrasies due to the small number of trials and the stimuli developed for this study. As such, additional research with a greater number of trials is needed to increase the external validity of the study design and gain a more accurate account of how participants would conduct their veracity judgments in real-world situations. It remains possible that differences in adults’ perceptions of how honest or dishonest Black children are compared to White children may indirectly contribute (at least in part) to many of the social inequalities that Black children face, but additional research is needed to achieve a better understanding of this possibility, including studies involving contexts where the consequences of incorrect veracity judgments are more severe. For example, Black children are particularly vulnerable in legal situations (as victims, suspects, or witnesses), where they may be susceptible to being kept in an unsafe environment (e.g., due to false denials of abuse) or being wrongfully convicted of a crime (e.g., due to false allegations or false confessions). For these reasons, it is especially important to identify factors that influence adults’ ability to make unbiased veracity judgments. Although this study investigates racial bias in veracity judgments within relatively low-stakes contexts compared to those that take place in a legal setting, it provides a foundation for future research to investigate veracity judgments of children’s reports in a variety of contexts.

## 5. Conclusion

To the best of our knowledge, this study is the first to identify systematic differences in adults’ veracity judgments of children’s simple denials of minor transgressions based on the race of both the child and the adult perceiver. Adult participants from both racial groups exhibited a truth bias in their veracity judgments of Black children, but not when evaluating the deceptiveness of White children. Consistent with the prejudice-related concerns hypothesis, the observed truth bias toward Black children was moderated by individual differences in participants’ desire to respond without prejudice, providing the first evidence of racial bias and prejudice-related concerns as potential barriers to making veracity judgments of children’s denials of a misdeed.

## Data availability statement

The original contributions presented in the study can be accessed at https://doi.org/10.17605/OSF.IO/983GB. Further inquiries can be directed to the corresponding author.

## Ethics statement

The studies involving human participants were reviewed and approved by the University of Regina Research Ethics Board. The patients/participants provided their written informed consent to participate in this study.

## Author contributions

SZ conceived the idea, secured funding, performed statistical analyses, and wrote the initial manuscript, with significant contributions by SH. SZ, SH, and LM contributed to the design of the study. SH collected the data. All authors contributed to revisions and interpretations.

## Funding

This research was supported through research grants awarded to the first author by the (1) American Psychology Law Society Grants in Aid for Early Career Professionals, and the (2) Luther College President’s Research Fund.

## Conflict of interest

The authors declare that the research was conducted in the absence of any commercial or financial relationships that could be construed as a potential conflict of interest.

## Publisher’s note

All claims expressed in this article are solely those of the authors and do not necessarily represent those of their affiliated organizations, or those of the publisher, the editors and the reviewers. Any product that may be evaluated in this article, or claim that may be made by its manufacturer, is not guaranteed or endorsed by the publisher.
